# Non-coding RNAs in Intercellular and Systemic Signaling

**DOI:** 10.3389/fpls.2012.00141

**Published:** 2012-07-05

**Authors:** Palmiro Poltronieri, Angelo Santino

**Affiliations:** ^1^Department of Biology and Agriculture, CNR-ISPA, National Research Council of ItalyLecce, Italy

## The RNA World

At the beginning of the last decade, it was believed that animal and plant genomes encoded, on average, 30,000 genes, surrounded by junk non-coding sequences. The dogma “from DNA to RNA to proteins” assigned RNA the role of transducer of genetic information into proteins. Large-scale studies have demonstrated that non-protein-coding RNAs are a massive output of transcription and can be regarded as regulatory RNAs. Following this new understanding, DNA is a static element of genetic information, a hardware, while RNA, in the form of multiple species of short and long non-coding transcripts constitutes the active information, the software (Hayashizaki and Carninci, 2006; Pang et al., 2007). The ability of RNAs to orchestrate chromatin states, DNA transcription, differential splicing, RNA translation, post-transcriptional modification, and protein stability determines a hidden layer of complexity of genetic information.

At the end of the last decade, microRNAs were identified by their role in regulation of proliferation and programmed cell death during the developmental stages of *Caenorhabditis elegans* (Grishok et al., 2001). In both plants and animals, microRNAs negatively affect their targets through a variety of transcriptional and post-transcriptional mechanisms.

Small RNAs (sRNAs) in plants include microRNAs, siRNAs (small interfering RNAs), tasi-RNA (trans-acting siRNAs), and nat-siRNAs (natural antisense-mediated siRNAs). The presence of methylated 3′ OH ends caused an under-representation of sRNAs in the analysis of RNA composition in plants, producing a bias in RNAs that were sequenced using tag sequencing and next-generation sequencing methods.

Today, sRNAs include an expanding number of 20–40 nt RNAs that function in the regulation of gene expression by affecting mRNA decay and translational inhibition, or lead to DNA methylation and gene silencing. They generally involve double-stranded RNA or stem loops and imply transcriptional or post-transcriptional gene silencing (PTGS). Gene silencing in plants can be mediated by siRNA, microRNA, tasi-RNAs, and nat-siRNAs. These RNAs act as single-stranded filaments incorporated into the effector complexes (RISC, RNA-induced silencing complex) containing one of the Argonaute enzymes degrading the target RNA complementary to sRNA (Bartel, 2009). DNA-dependent RNA polymerase IV (Pol IV)-dependent sRNAs are 24-nt and associated with AGO4, whereas the majority of the potential Pol V-dependent sRNAs are 21-nt and bound to AGO1, suggesting the potential involvement of AGO1 in Pol V-related pathways (Dunoyer et al., 2010a,b; Wang et al., 2011a,b). Their role is in the amplification of signals such as RNA-primed RNA amplification, and in the spread of anti-viral RNA interference mechanisms.

In higher plants, a number of physiological processes are regulated by the transfer of signaling molecules through the phloem. This phloem-mediated remote-control system provides specific and efficient regulation to fine-tune many plant developmental programs. In addition to hormones, proteins, and small peptides, phloem is rich in RNA-binding proteins that function as chaperones for systemic RNA movement.

In 2004, an international consortium within the EU VI Framework Program, “Riboreg”^1^, supported the production of the first *Arabidopsis* non-coding RNA macroarrays, that characterized their regulation in different tissues and in plants subjected to environmental stresses (Laporte et al., 2007; Bardou et al., 2011).

Massively Parallel Signature Sequencing (MPSS, Lynx Technologies) was used before the advent of next-generation sequencing as a high-throughput method to identify short transcripts devoid of poly-A tails, as microRNAs and sRNAs (Sunkar and Zhou, 2004). In September 2005, Science published a monograph on non-coding RNAs the first analysis of a large number of small transcribed *Arabidopsis* RNAs using MPSS (Lu et al., 2005).

In this timeframe, findings on viroids and the movement of viral proteins through the phloem induced a revision of the phloem function. Viruses and other macromolecules are transported through plasmodesmata. Virus movement requires virus-encoded proteins that interact with host factors. Different strategies of movement are exploited by different viruses. These mechanisms were recently reviewed (Atkins et al., 2011).

## RNA Complexes Moving in the Phloem

It appeared evident that RNA-binding proteins in phloem were important in facilitating the movement of RNA interference mediators. Comprehensive analyses of RNA molecules present in the phloem sap were carried out (Yoo et al., 2004; Aoki et al., 2005; Omid et al., 2008; Burgyan and Havelda, 2011). Scientists further identified phloem-specific RNA-binding proteins (19) and a large population of sRNAs including known microRNAs and various endogenous siRNA species (20). For instance, phloem small-RNA-binding protein 1 (PSRP1) was subsequently shown to bind and facilitate movement of single-stranded sRNA molecules between cells (Ham et al., 2009). CmPP16 protein from *Cucurbita maxima* was shown to possess properties similar to those of viral movement proteins (Aoki et al., 2005). Plants deploy RNA silencing, *R*-gene-mediated defense and other mechanisms to prevent phloem transport of viruses. Plant viruses use sieve elements in phloem as the route of long-distance movement and systemic infection in plants, and viral RNA-binding proteins are exploited by viruses to counteract the plant RNA silencing machinery (Lakatos et al., 2004; Zhang et al., 2009). Thus, phloem is like a highway that is able to target macromolecules across plant tissues through specific cargo proteins (Takeda and Ding, 2009; Cao et al., 2010) and influences the systemic spread of silencing (Vuorinen et al., 2011). Voinnet and Baulcombe independently contributed to the understanding of RNA silencing, especially in the anti-viral response, by reviewing the difference in the two mechanisms of extensive local spread and system signaling in RNA interference (Voinnet and Baulcombe, 1997; Baulcombe, 2004; Brosnan and Voinnet, 2011; Melnyk et al., 2011). Silencing spreads systemically through the phloem system of the plants, which also translocates metabolites from source to sink tissues. Grafting experiments were essential to allow a clear separation between the silencing signal-producing (source) tissues and the signal-receiving (sink) tissues (Kalantidis et al., 2008). Unlike the short-range silencing signal, there is still little knowledge on the mediators of systemic silencing, which is possibly a cooperative activity mediated by different RNA molecules. Whatever the identity of the systemic signals (siRNA precursors with size ranging form 22 to 40 nt), it is assumed to move through the phloem (Baulcombe, 2004; Moissiard et al., 2007). This was confirmed by studies using a phloem flow tracer (Tournier et al., 2006), which also allowed for a more detailed analysis of the specific properties of RNA spread.

## The Evolving Roles of RNA Sequences Acting at Distance

The literature on phloem as a highway for macromolecules is expanding (Lough and Lucas, 2006; Chuck and O'Connor, 2010; Chitwood and Timmermans, 2010; Dinant and Lemoine, 2010). While RNA sequencing and bioinformatic analysis have provided information on microRNAs present in plant phloem (Buhtz et al., 2008; Deeken et al., 2008), several microRNAs have been shown to move through the phloem to exert their activity at a distance. For example, miR166 was shown to act at distance and to induce maize leaf polarity (Juarez et al., 2004), and miR172 is present in sRNA libraries made from phloem exudates, acting as part of a long-distance signaling network (Zeevaart, 2008). It moves between tissues upon grafting and promotes tuberization in potato (Martin et al., 2009). On the other hand, miR319 moves from leaves to roots where it targets a subset of the *TCP* family of transcription factors that regulates *LOX2* expression (Yoo et al., 2004; Schommer et al., 2008; Buhtz et al., 2010).

Phloem-specific sRNAs were also found to travel and inform the roots of the nutrient status of the shoot. The levels of three miRNAs known to respond to nutrient deprivation in non-vascular tissue, miR395 (sulfate), miR398 (copper), and miR399 (phosphate), were increased in phloem sap during the growth of plants under the respective starvation conditions (Buhtz et al., 2008). Plants regulate inorganic phosphate (Pi) homeostasis with cross-talk between roots, stem, and leaves to adapt to environmental changes in Pi availability. During phosphate-limitation conditions, plants respond with increased phosphate uptake from the soil and phosphate mobilization from the leaf. Upon Pi starvation, miR399 is synthesized in leaves and travels along the phloem to the roots, where it cleaves its target gene, PHO2, in *A. thaliana*, a ubiquitin-conjugating E2 enzyme, thereby releasing several protein targets from ubiquitinylation-dependent degradation and increasing Pi content in the shoots (Franco-Zorrilla et al., 2007). Inhibition of miR399 causes depletion of phosphate in the leaves. MicroRNAs become unable to bind to target mRNAs for the presence of RNA mimics possessing similar target sequences. IPS1 (induced by phosphate starvation1) RNA modulates the availability of miR399 sequestering miR399 releasing from inhibition *PHO2* mRNA. A similar mechanism of “target mimicry” was proposed for the anti-mir sponges, able to sequester and inactivate complementary microRNAs. Artificial target mimic sites in plants could indeed reduce translation efficiency in *cis* (Todesco et al., 2010).

Several messenger RNAs travel a long-distance in the phloem, such as *BEL5*, *LeT6*, *KNOTTED1*, *DELLA-GAI*, and *FLOWERING LOCUS T* (*FT*) mRNA (Kehr and Buhtz, 2010). *FT* is a signal molecule that acts at a distance, producing flowering in the leaf meristems to induce floral development. FT protein may require *FT* mRNA together with phloem shuttle proteins to form a complex that is transported through phloem translocation stream to the shoot apical meristem (SAM). This was shown using non-conventional approaches that exploit virus-induced RNA silencing and meristem exclusion of virus infection (Li et al., 2009, 2011). Thus, from the *FT* mRNA structure and from other RNA sequences, it was shown that the tertiary structure is important to define their function (Zhong et al., 2007; Chitwood and Timmermans, 2010).

## Challenges for the Next Studies

Omics and bioinformatics are essential to understanding the molecular systems that underlie various plant functions. Recent sequencing technologies have revitalized sequencing approaches in genomics and have produced opportunities for various emerging analytical applications. A new EU FP7 project now started, AB-Stress, will study the epigenetic regulation and the stress induced sRNA world in legumes during biotic and abiotic stresses^2^. Several new-omics layers such as the interactome, epigenome, peptidome, and hormonome are taking the lead that will open new perspectives on the complex regulation of communication between plant tissues and in signaling at a distance.
